# A Case Reflecting the Impact of a Deferred Transesophageal Echocardiogram on a COVID-19 Disease Patient

**DOI:** 10.7759/cureus.33007

**Published:** 2022-12-27

**Authors:** Kofi Seffah, Walter Y Agyeman

**Affiliations:** 1 Internal Medicine, Piedmont Athens Regional, Athens, USA

**Keywords:** vaccine, covid-19, echocardiogram, policies, procedures

## Abstract

COVID-19 has been amongst us for over two years and has seen a strong response from the international health community regarding mobilization. There has also been the advancement of vaccine science-regulations surrounding the day-to-day decisions regarding patients who tested positive for the virus. Guidelines for treatment continue to vary. While this may benefit most patients, more objective metrics in deciding elective, urgent, and emergent procedures have been proposed. In this paper, a patient who tested positive for the virus was refused a procedure ( transesophageal echo ) based on COVID status, which may have contributed to a delay in treatment and detrimental outcomes.

## Introduction

Following the WHO declaration of the COVID pandemic in 2020, various facets of the healthcare wheel were instructed to adjust to the growing need for protecting patients, health personnel, and the general public. One such decision, amongst some practices, was to defer surgical procedures on the grounds of the potential risk for infection of operation staff, with the risk being higher than the benefit of the surgery to the one patient. Since then, there has been a decline in the virus's spread following the vaccine's introduction. There, however, remains an absence of that unifying consensus on what constitutes a safe surgery or high risk amongst patients in whom the diagnosis is made.

Below is a case report of a patient who stood to benefit from a transesophageal echo on the grounds of embolic showers found on her MRI. The procedure was, however, deferred, citing her COVID-19 status, amongst other reasons, as the reason not to proceed [1,2]. The question we seek to address is, 'Does a procedure needed in urgent need to be deferred based on a provider's discretion in the setting of COVID-19 disease, or will parameters and more precise guidelines are beneficial in future management decision-making?

## Case presentation

A 65-year-old female with a history of hypertension was admitted to the hospital on February 11, 2022, with an altered mental status. She presented with no cardiovascular or respiratory symptoms. However, she had previously tested positive for COVID-19 from the referral facility on February 3, and no need for treatment had been established. Her main presentation was decreased responsiveness, with waxing and waning levels of consciousness. She had been vaccinated against COVID-19 (the second dose of the Moderna vaccine received in November 2021 ) and influenza by admission.

Her vital signs were stable on arrival. Initial work-up demonstrated thromboembolic phenomena as evidenced on head CT, with areas of hypoperfusion suggestive of a stroke, and was subsequently diagnosed with Cryptococcal meningitis by CSF PCR. Initial evaluation by the neurology team was that though the diagnosis of Cryptococcal meningitis had been rightly made (supported by leptomeningeal enhancement on MRI) and could substantively account in part for the clinical presentation, her imaging findings of bilateral diffuse emboli in multiple cerebellar territories on MRI, were not characteristic of the thromboembolism caused by the organism, Cryptococcus.

**Figure 1:**
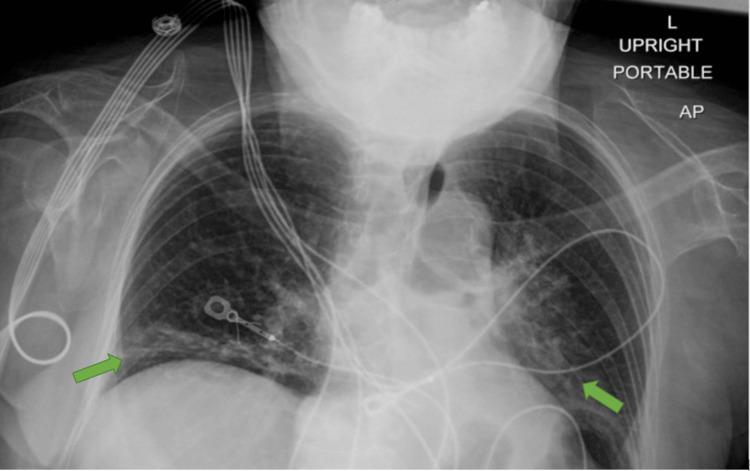
X-ray indicating bibasal atelectasis. Green arrows indicate areas of bilateral atelectasis along the diaphragmatic margins.

Another cause of her embolic event had to be investigated. A chest x-ray (figure 1) showed patchy bibasilar opacities, likely atelectasis, with a low likelihood of pneumonia. Bilateral carotid artery dopplers showed no significant stenosis. A transthoracic echo proved unrevealing, showing a hyperdynamic left ventricular ejection fraction of 70% and a grade I diastolic dysfunction with no noted valvulopathies, and there was an initial move to go further with a transesophageal echocardiogram, with the view to exposing potential valvular lesions-this required cardiology input at this point.

Though agreeing with the assertions of their neurology counterparts, the cardiology consult opted to hold back on the more invasive approach, citing, amongst other things, her COVID-19 positivity status as a limiting factor. The patient was started on anticoagulation, and further evaluation was deferred.

This patient went on to experience deleterious CNS sequelae of the presumptive diagnosis of Cryptococcal meningitis, requiring several lumbar punctures, antifungal treatment with Amphotericin B and flucytosine, later showing no recovery of baseline neurological status. Her GCS remained below 8 by her discharge to palliative care. She was discharged on February 25 to a nursing home. On discharge, it was unknown whether her entire presentation was due to a single disease process or if there were overlapping conditions, including COVID-19-related embolic phenomena. Her prognosis remained guarded at the time of discharge.

## Discussion

The first case of COVID-19 disease was documented in the Wuhan district of China in December 2019 [1]. The WHO declared a pandemic on March 11, 2020 [2]. The basic reproduction number [k1] R-naught (defined as the average number of people typically infected by an index case for the original stain of the disease as noted in Wuhan, was between 2 and 3 [3]. Subsequent virus strains have been noted to have higher R-naughts, between 5 and 8, particularly the Delta strain [4]. This, however, does not necessarily correlate with virulence ( defined as the severity of harm from the disease ). A person who tested positive, with a determined high basic reproduction number, may only be assumed to be potentially spreading a virulent strain. 

COVID-19 disease has been associated with thromboembolic phenomena and could have accounted for findings on brain imaging in our patient [5]. It has also been associated with prolonged periods of clinical and subclinical sequelae, lasting beyond, in some cases, 3 months of initial positive testing, which, in part, may be responsible for the lethargy she presented with [6]. Thromboembolic phenomena from Cryprococcal infection are marked by small vessel vasculitis typically affecting the basal ganglia and thalamus [7], and this was not seen in our patient. 

The evaluation of our patient for valvulopathy, abscess, or endocardial compromise may have pointed to an alternate line of treatment besides anticoagulation. She may have developed an abscess or vegetation not picked up on the transthoracic echo.

Guidelines for managing COVID-19 were first proposed by the WHO and the CDC in 2020, mainly targeting public safety through the prevention of spread [2,8]. More nuanced decision-making about the management of patients infected with pneumonia has evolved, seeing various treatment modalities progress from anecdotal ( ivermectin, quinine ) to mainstream ( steroids, monoclonal antibodies ) over the period [9]. The association of the disease with other co-morbidities, such as pulmonary embolism, increased risk of thrombus formation, and increased susceptibility to respiratory illness, may persist beyond the hospitalization period. Daily decisions requiring further and better recommendations continue to emerge in clinical practice. On March 13, 2020, the American College of Surgeons proposed elective rescheduling procedures and shifting urgent elective procedures to the outpatient setting. In November 2020, a joint statement was released by the American College of Surgeons, American Society of Anesthesiologists, Association of perioperative Registered Nurses, and the American Hospital Association updating the aforementioned to include guidelines for physicians, surgeons, perioperative and nursing staff [10]. However, these guidelines referenced reliance on the CDC for further particulars or details. A large portion of decision-making was also left to the practitioner. In cases where urgency was required, these guidelines recommended using objective decision-making tools, including the MeNTS instrument - which is medically necessary and time-sensitive [11].

These measures are seen as largely preventative, noting that they were employed at the onset of the pandemic, when there was more to be known about the virus. Also, the above guidelines were designed with clinically active disease in mind. However, milder, insidious forms of the illness have been reported, such as the post-COVID disease, which cannot be accounted for except by a positive COVID test and suggestive history [3].

The absence of universalization of these tools and guidelines has seen some patients in various health institutions receive airborne precautions for a positive test and no symptoms. Others receive no precautionary measures despite known positive status in the ambulatory setting. The MeNTS tool on our patient showed likely low to moderately low scores and a low likelihood of causing harm if her TEE was carried out. Indeed, this may have brought a greater degree of closure and more rapid treatment and may have changed the trajectory of her outcome.

COVID-19 disease has seen an advancement in vaccine science and the development of mRNA vaccines, including the Moderna, Pfizer, and AstraZeneca vaccines [12]. The global impact has subsided, and although COVID-19 remains of high public health importance, much of the stigma surrounding the virus has been elucidated [13,14].

## Conclusions

Due to its mode of spread (airborne ), the decision for elective procedures in the setting of positive testing for the condition has remained mainly at the practitioner's discretion. Guidelines initially focused on clinically active COVID-19 illness. In the case of emergent procedures in non-symptomatic carriers, such as the recommended TEE in our patient, we recommend a spelled-out approach to protect the interests of the patient, protect practitioners from litigation, prepare resources for better and more definitive management and enable more confident documentation on what now seems to be a grey area on the matter. This may be lifesaving. The stigma surrounding the disease and limitations to treatment have been reduced with better understanding and the arrival of vaccination. This calls for practice and guidelines to be developed, evolved, and followed.
